# Implantable cardioverter-defibrillators in congenital heart disease

**DOI:** 10.1007/s00399-016-0437-3

**Published:** 2016-06-01

**Authors:** H. Chubb, E. Rosenthal

**Affiliations:** Department of Paediatric Cardiology, Evelina London Children’s Hospital, Westminster Bridge Road, SE1 7EH London, UK; Division of Imaging Sciences and Biomedical Engineering, King’s College London, London, UK; Adult Congenital Heart Disease Group, Departments of Cardiology at Guy’s and St Thomas’ NHS Foundation Trust & Evelina London Children’s Hospital, London, UK

**Keywords:** Implantable cardioverter-defibrillator, Congenital heart disease, Ventricular tachycardia, Sudden cardiac death, ICD, Angeborene Herzfehler, Ventrikuläre Tachykardie, Plötzlicher Herztod

## Abstract

Implantable cardioverter-defibrillators (ICD) have an important role in reducing sudden cardiac death in patients with congenital heart disease (CHD); however, the benefit of ICDs needs to be weighed up against both short-term and long-term adverse effects, which are difficult to evaluate in the heterogeneous CHD population. A tailored approach, taking into account risk stratification and patient-specific factors, is needed to select the most appropriate strategy. This review discusses primary and secondary ICD indications, implantation approaches and long-term follow-up. Recent publications have shed light on the concerns of system longevity, lead extractions, inappropriate shocks and impact on the quality of life. All of these factors require consideration prior to commitment to this long-term treatment strategy.

## Zusammenfassung

Implantierbare Kardioverter-Defibrillatoren (ICD) spielen bei Patienten mit angeborenen Herzfehlern (AHF) eine entscheidende Rolle bezüglich der Reduktion des plötzlichen Herztods. Die Vorteile einer ICD-Implantation müssen jedoch den potenziellen akuten, aber auch langfristigen Komplikationen gegenübergestellt werden, was insbesondere bei der Gruppe der AHF-Patienten schwierig ist. Individuelle Strategien, die den patienten-spezifischen Faktoren einerseits und der notwendigen Risikostratifizierung andererseits Rechnung tragen, müssen sorgfältig erarbeitet werden. In diesem Übersichtsartikel werden die ICD-Indikationen zur Primär- und Sekundärprophylaxe, die Implantationstechniken und die Ergebnisse aus der langfristigen Nachbeobachtung diskutiert. Daten aus aktuellen Studien belegen spezifische Limitationen bezüglich Haltbarkeit der Systeme, Umsetzbarkeit von Sondenextraktionen, inadäquaten Schockabgaben und Reduktion der Lebensqualität. Alle diese Faktoren müssen sorgsam abgewogen werden, bevor bei Patienten mit angeborenen Herzfehlern über eine ICD-Implantation als langfristige Behandlungsstrategie entschieden wird.

## Introduction

Sudden cardiac death (SCD) is a major cause of mortality in the congenital heart disease (CHD) population [40]. A large proportion of these deaths are caused by ventricular arrhythmias that may be amenable to timely cardioversion by implantable cardioverter-defibrillators (ICD); however, the selection of patients, mode of implantation and long-term management of the devices in the CHD population is challenging. In such a heterogeneous patient group, robust evidence-based guidelines are flexible by definition. This review discusses these concerns in the light of recent publications.

## Indications

**Fig. 1:**
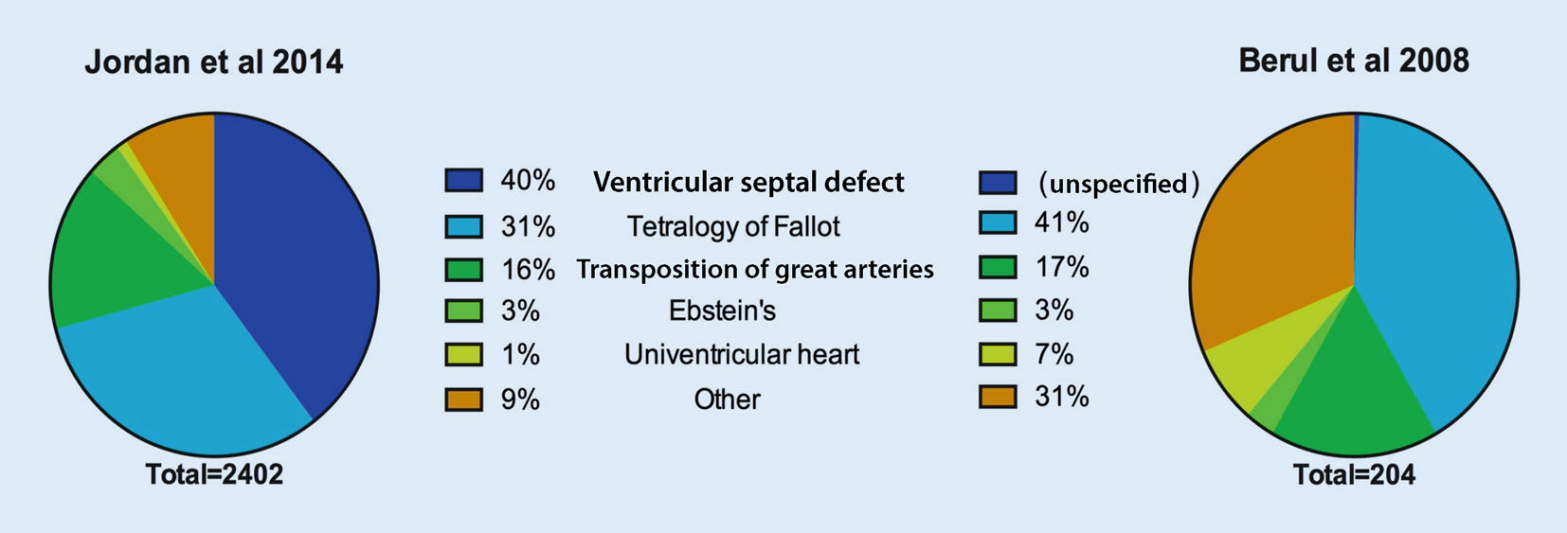
Distribution of congenital heart disease (CHD) implantable cardioverter-defibrillator population by CHD lesion. Figures are adapted from the two largest population studies. The 1304 patients reported by Jordan et al with atrial septal defect (ASD) alone are excluded. No patients with VSD alone were reported by Berul et al [3, 13]

The CHD population represents a very small minority of ICD implantations but has been relatively well-defined in registries and thorough meta-analyses [1, 13, 39]. There is a surprisingly wide variance in composition of CHD ICD cohorts, which is likely to reflect both coding as well as clinical practice ([3, 13, 25], Fig. 1).

Consensus guidelines for arrhythmia management in adult CHD [18] and paediatric [4] populations have been published, establishing the broad indications for ICD implantation in the CHD cohorts and these should be interpreted in the context of the 2008 American Heart Association (AHA) device guidelines and the 2015 European Society of Cardiology (ESC) guidelines on the management of ventricular arrhythmias [10, 42].

### Secondary prevention

The role of ICD implantation in CHD patients following resuscitation for cardiac arrest is usually self-evident and in the absence of a clearly reversible cause this is a class 1 (level B) indication (Tab. 1). The implantation of ICD is also recommended for symptomatic and sustained ventricular tachycardia (VT), in the absence of loss of cardiac output, following detailed evaluation (class 1, level B).

**Tab. 1:** Recommendations for management of ventricular arrhythmias in patients with congenital heart disease. Adapted from the 2015 European Society of Cardiology guidelines for the management of patients with ventricular arrhythmias (Priori et al.) [42]

Class	Clinical indication	Level of evidence
Class I	After evaluation to define the cause of the event and exclude any reversible causes, ICD implantation is recommended for patients with CHD who are survivors of an aborted cardiac arrest	B
	ICD implantation is recommended for patients with CHD with symptomatic sustained VT who have undergone haemodynamic and electrophysiological evaluation	B
	Catheter ablation is recommended as additional therapy or an alternative to ICD in patients with CHD who have recurrent monomorphic VT or appropriate ICD therapies that are not manageable by device reprogramming or drug therapy	C
	ICD therapy is recommended in adults with CHD and a systemic LVEF < 35 %, biventricular physiology, symptomatic HF despite optimal medical treatment and NYHA functional class II or III	C
Class IIa	ICD implantation should be considered in patients with CHD with syncope of unknown origin in the presence of either advanced ventricular dysfunction or inducible sustained VT or VF on PVS	B
	ICD implantation should be considered in selected patients with tetralogy of Fallot and multiple risk factors for SCD, including LV dysfunction, non-sustained VT, QRS duration > 180 ms or inducible sustained VT on PVS	B
	Catheter ablation should be considered as an alternative to drug therapy for symptomatic sustained monomorphic VT in patients with CHD and an ICD	B
Class IIb	ICD therapy may be considered in patients with advanced single or systemic RV dysfunction in the presence of other risk factors such as non-sustained VT, NYHA functional class II or III or severe systemic AV valve regurgitation	B
	PVS may be considered for risk stratification of SCD in patients with tetralogy of Fallot who have one or more risk factors among LV dysfunction, non-sustained VT and QRS duration > 180 ms	B
	PVS may be considered in patients with CHD and non-sustained VT to determine the risk of sustained VT	C
	Surgical ablation guided by electrophysiological mapping may be considered in patients with CHD undergoing cardiac surgery, with clinical sustained VT and with inducible sustained monomorphic VT with an identified critical isthmus.	C
Class III	PVS is not recommended to stratify the risk in patients with CHD in the absence of other risk factors or symptoms	C

*AV * atrioventricular, *CHD* congenital heart disease, *HF *heart failure, *ICD* implantable cardioverter-defibrillator, *LV* left ventricle, *LVEF* left ventricular ejection fraction, *NYHA* New York Heart Association, *PVS* programmed ventricular stimulation, *RV* right ventricle, *VF* ventricular fibrillation, *VT* ventricular tachycardia

In a small subgroup of patients with sustained VT, the option of catheter ablation alone is tantalising. Small studies have demonstrated high rates of long-term VT-free survival in patients for whom a critical isthmus is transected via catheter ablation [28, 43], particularly in those who remain recurrence-free after the first 2 months postablation. There may be a place in a highly selected patient group for management without ICD implantation [10], possibly with short-term management with a wearable defibrillator but a more detailed evidence base is required to engender confidence in such an approach.

### Primary prevention

Approximately half of all ICD implantations in CHD are for primary prevention [3, 13, 25, 39] and the indications for this cohort remain uncertain. The overall appropriate shock rate is highly dependent on the indications but occurs in approximately 1 in 5 patients over the first 3 years postimplantation [39], a surprisingly high proportion compared to the non-CHD population; however, this figure needs to be weighed up against the relatively high ICD-related complication rate of 1 in 4 and the additional inappropriate shock rate of 1 in 4, both over 3 years [1, 39, 41]. Furthermore, appropriate shocks do not always indicate aborted death and the balance of risk and benefit therefore requires careful evaluation.

The indications for primary prevention are most frequently non-sustained VT, impaired systemic ventricular function and syncope. Symptomatic non-sustained VT and subpulmonary ventricular dysfunction have been shown to be associated with appropriate shocks [25]. The role of programmed ventricular stimulation (PVS) studies remains controversial with conflicting evidence, but this may reflect to some degree the different patient substrate. Surprisingly and importantly, a meta-analysis showed that the presence of inducible VT does not predict appropriate interventions (odds ratio 1.2, range 0.2–5.7) [39].

#### Tetralogy of Fallot

The most detailed data for primary prevention ICD in CHD relates to those with repaired tetralogy of Fallot (ToF). For these patients, the risk of SCD is approximately 2–3 % per decade [42] and a number of risk factors have been identified that delineate increased risk and therefore the group that will derive greatest benefit from ICD implantation (Tab. 2). Raised left ventricular end-diastolic pressure (LVEDP), pulmonary artery pressure and right ventricular (RV) systolic pressure are the strongest predictors of appropriate shock therapy. The significance of inducible sustained VT on PVS is unclear, with a trend towards increased incidence of appropriate shocks in inducible patients [20, 24]. Retrospective studies have reported appropriate shock rates as high as 17 % per year for high-risk primary prevention (risk score 6–12) but with an inappropriate shock rate of 5.8 % per year and other system complications occurring in nearly 30 % [20].

**Tab. 2:** Predictors of appropriate implantable cardioverter-defibrillator therapy in primary preventative therapy of tetralogy of Fallot (0–2 points is low risk, 3–5 points intermediate risk and 6–12 points high risk. Adapted from Khairy et al. [20]). Significant factors on univariate and multivariate analysis are highlighted in italics

	HR	95 % CI	Univariate analysis	Multivariate analysis	Points attributed
Prior palliative shunt	2.6	0.7–9.4	0.13		2
Inducible sustained VT	2.1	0.6–7.6	0.24		2
QRS duration, per 1 ms	1.01	0.99–1.03	0.21		
QRS ≥ 180 ms	2.0	0.7–5.9	0.22		1
Ventriculotomy incision	2.4	0.9–6.1	0.071		2
Nonsustained VT	2.7	1.0–7.2	0.053	*0.023*	*2*
RV systolic pressure, per 1 mmHg	1.06	1.01–1.11	*0.0301*		
Mean PAP, per 1 mmHg	1.16	1.05–1.35	*0.0032*		
LVEDP, per 1 mmHg	1.21	1.08–1.35	*0.0008*	*0.0039*	
LVEDP ≥ 12 mmHg	15.1	1.9–123.7	*0.0114*	*0.022*	*3*

*HR* hazard ratio, *CI* confidence interval*, VT* ventricular tachycardia*, PAP* pulmonary artery pressure*, LVEDP* left ventricular end diastolic pressure, *RV* right ventricle

#### Failing systemic ventricle

The use of ICD should be considered in patients who meet the established class I indications for the failing left ventricle, including LV ejection fraction < 35 % with New York Heart Association (NYHA) class II or III symptoms ([10, 42], Tab. 1); however, only a very small proportion of patients with CHD will meet these criteria and the indications for ICD implantation in the context of systemic ventricular dysfunction are much more nuanced.

Proponents [36] and antagonists [38] have made well-reasoned arguments for and against the routine role of ICD in the failing systemic ventricle, particularly as the ejection fraction falls below 30 %. The applicability of the major non-CHD ICD trial data to a CHD cohort is highly questionable, with very different pathophysiology and risk-benefit profiles. There are certainly selected patient groups that stand to have lives saved through appropriate therapies. The identification, though, of these patients requires further investigation.

#### Atrial switch

In patients with transposition of the great arteries (TGA) treated by an atrial switch procedure (Mustard or Senning), the risk of SCD is approximately 5 % per decade of life and atrial arrhythmias appear to be a strong predictor of events [15]; however, appropriate shock rates for primary prevention ICDs are lower in this patient group than ToF, at approximately 0.5 % per year and the indications should be carefully considered [16].

Risk factors for appropriate therapies are thought to include wide QRS duration, systemic atrioventricular (AV) valve regurgitation and systemic right ventricular (RV) dysfunction. Systemic RV function cut-off values have been difficult to define but are likely to be lower than the 35 % for LV dysfunction and should be weighed up against relatively high complication rates (14 out of 37 TGA patients [38 %] in the largest cohort study [19]). PVS does not appear to be a useful predictor of events.

#### Univentricular heart

Patients with univentricular circulation differ widely in terms of underlying cardiac morphology and subsequent surgical strategy and data on the efficacy of ICD therapy is scarce. Atrial arrhythmias are common but there is also a significant incidence of arrhythmia-related SCD, reported to be up to 10 % over 10 years [17]. Most ICD in this patient group are implanted for secondary prevention or in the context of severe univentricular systolic dysfunction. Formal risk factor stratification is lacking and the role of PVS is unknown.

## Implantation

Transvenous ICD systems make up the vast majority of implantations in the largest registries (typically 97 % of the paediatric and CHD population [13]). Transvenous implantation is generally felt to be preferable to non-transvenous on account of superior system longevity (system survival at 3 years 76 % versus 49 %, respectively [34]) and defibrillation efficacy (failure to cardiovert 0.2 % versus 3.7 %, respectively [13]). However, these advantages need to be weighed up against the requirement for lead(s) in the vascular system, subsequent lead extraction and high risk baffle punctures; therefore, in selected cases a non-transvenous system remains the most suitable option. A detailed understanding of cardiac anatomy and prior surgical and interventional procedures is mandatory, and frequently up to date imaging, including computed tomography (CT), magnetic resonance imaging (MRI) and venography are necessary to clarify the substrate before implantation. Placement of a lead into a systemic ventricle or in the presence of a right to left shunt is possible but subsequent long-term anticoagulation is indicated [4, 21].

### Transvenous systems

**Fig. 2:**
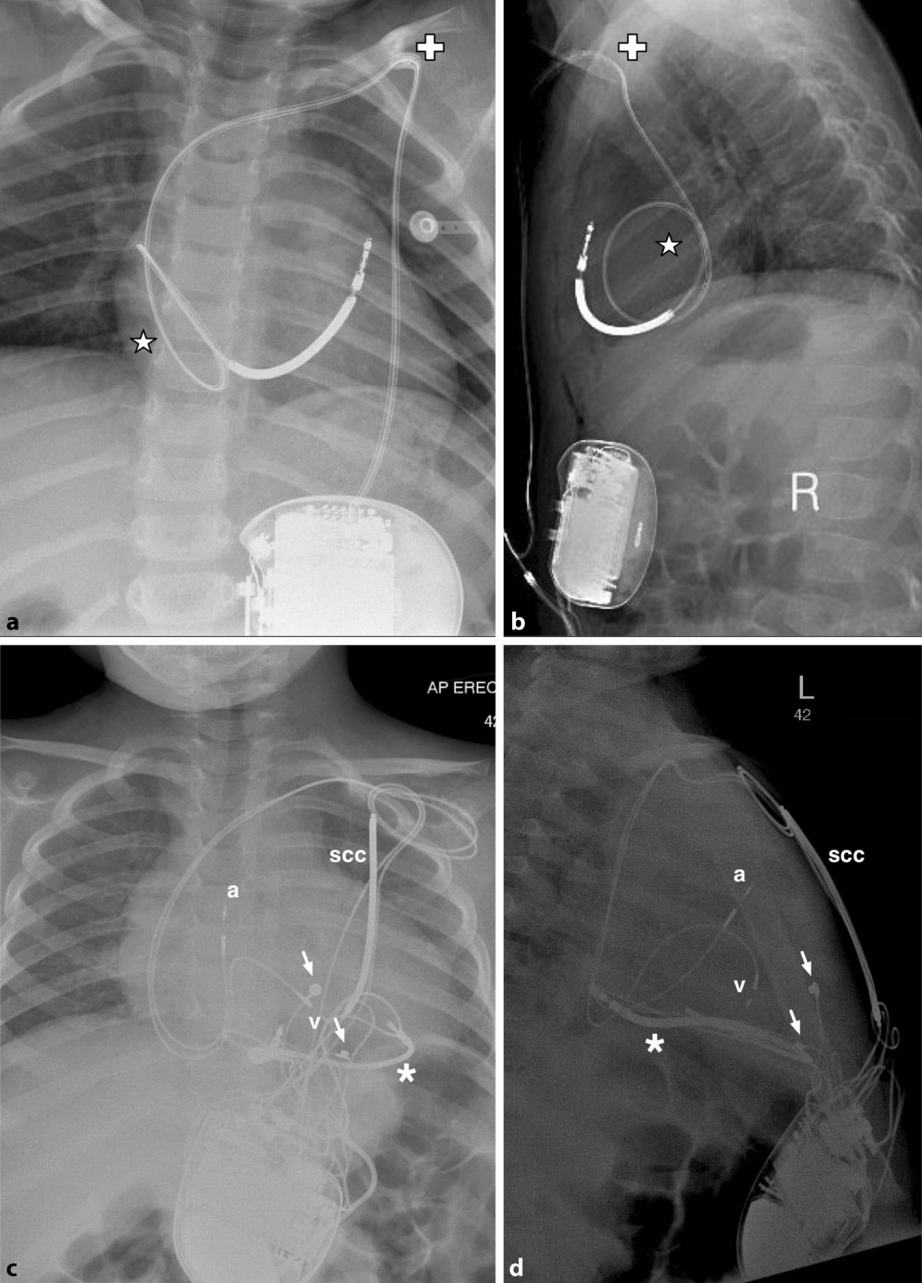
**a** Posteroanterior and **b** lateral radiographs demonstrating transvenous pacing system in an 18 kg child with long QT (LQT) syndrome. Generator has been placed abdominally with lead tunnelled to left subclavian vein (cross). Note redundant loop to accommodate growth (star). **c** Posteroanterior and **d** lateral radiographs demonstrating a more complex hybrid system in a 15 kg child with LQT type 3. The first ICD implanted was non-transvenous, with intrapericardial coil (asterisk) and epicardial sense and pacing leads (white arrows). Subsequent failure of the epicardial pacing leads with R‑wave undersensing and rising defibrillation threshold necessitated upgrade of the system with subcutanous coil (scc) and transvenous atrial (a) and ventricular (v) pace and sense leads (**c** and **d** courtesy of Jasveer Mangat, Great Ormond Street Hospital, London)

In adult practice, ICDs can be inserted in a subcutaneous or submuscular pocket with the patient under local anaesthesia with sedation, with all leads implanted transvenously. For children above 20–25 kg in weight, small ICDs can be implanted in a similar fashion under general anaesthesia but for those weighing 10– 20 kg an abdominal pocket for the generator should be considered, with leads tunnelled to the subclavian vein (Fig. 2a, b). Below 10 kg, a non-transvenous system should generally be considered ([4], Fig. 2c, d).

Modern lead sizes are only marginally larger than pacemaker leads but historically results for thin leads (≤ 7Fr) have been confounded by issues with lead failures and recalls for Sprint Fedelis (Medtronic, Minneapolis, MN) and Riata (St Jude, Little Canada, MN) leads. Smaller contemporary leads include the Durata (St Jude, 7Fr) and the Protego (Biotronik, Berlin, Germany, 7.8Fr) and long-term outcome data are awaited. In addition, younger age at implantation is associated with earlier lead failure [1].

In patients with difficult transvenous access or repaired complex congenital heart disease, innovative solutions may be required to facilitate transvenous lead deployment. The hemi-Fontan, Fontan and atrial switch anatomies provide particular challenges, but the placement of shock coils in a collateral or azygous vein [31] are alternative options.

#### Generator and system selection

**Fig. 3:**
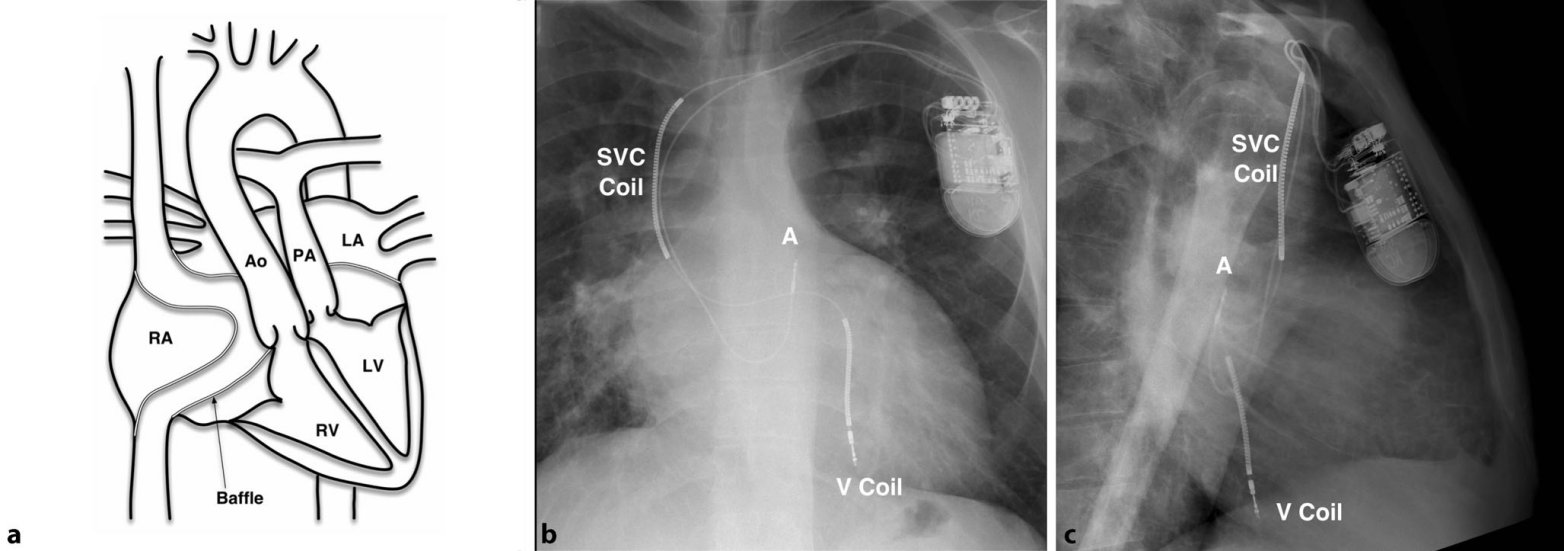
Diagram of transvenous implantable cardioverter-defibrillator implanted following Senning repair (patient aged 18 years) for transposition of the great arteries (**a**). Posteroanterior (**b**) and lateral (**c**) radiograph projections. Note ventricular coil (*V* *Coil*) placed in the posterior LV via the venous baffle. *A* atrial sense/pace lead, *SVC* superior vena cava, *RA* right atrium. *Ao* Aorta, *PA* pulmonary artery, *LA* left atrium, *RV* right ventricle and *LV* left ventricle

A single pace/sense ventricular lead, generally integrated within the shock lead, is mandatory for sensing and defibrillation and these single chamber systems are capable of bradycardia support and antitachycardia pacing (ATP) (Fig. 3). Evidence for selection of dual over single coil leads in the CHD population is limited and may complicate lead extraction. An atrial lead adds the capability of atrial bradycardia pacing, AV sequential pacing and atrial ATP. In theory, there should also be enhanced rhythm discrimination but a study of 168 CHD patients with single versus dual chamber ICD has suggested that there is no significant reduction in inappropriate shocks in the CHD patient group [27].

### Non-transvenous systems

Non-transvenous systems remain appropriate in a subgroup of patients, particular Fontan and atrial switch patients. In contrast to the subcutaneous ICD (S-ICD), these ICD systems remain capable of providing antibradycardia or antitachycardia pacing, and may also be deployed in very small patients. Generators are generally placed in the abdomen, with a shock coil placed in the left thorax (pericardial or pleural space, anteriorly or posteriorly) and placement of the ventricular lead epicardially ([5], Fig. 2c, d). Cardiac strangulation from epicardial coils in a growing child has not been reported as yet.

### Subcutaneous

There is increasing evidence for the use of subcutaneous ICD (Emblem S‑ICD, Boston Scientific, Boston, MA), avoiding the potential intravascular and lead complications of other systems. They are relatively bulky and do not allow for conventional pacing or antitachycardia pacing and therefore may generally be more appropriate for channelopathies and other arrhythmias not associated with structural CHD. Generator erosion rates in smaller patients are high, with 3 out of 7 children in one case series requiring reoperation for threatened erosion or wound dehiscence [12].

**Fig. 4:**
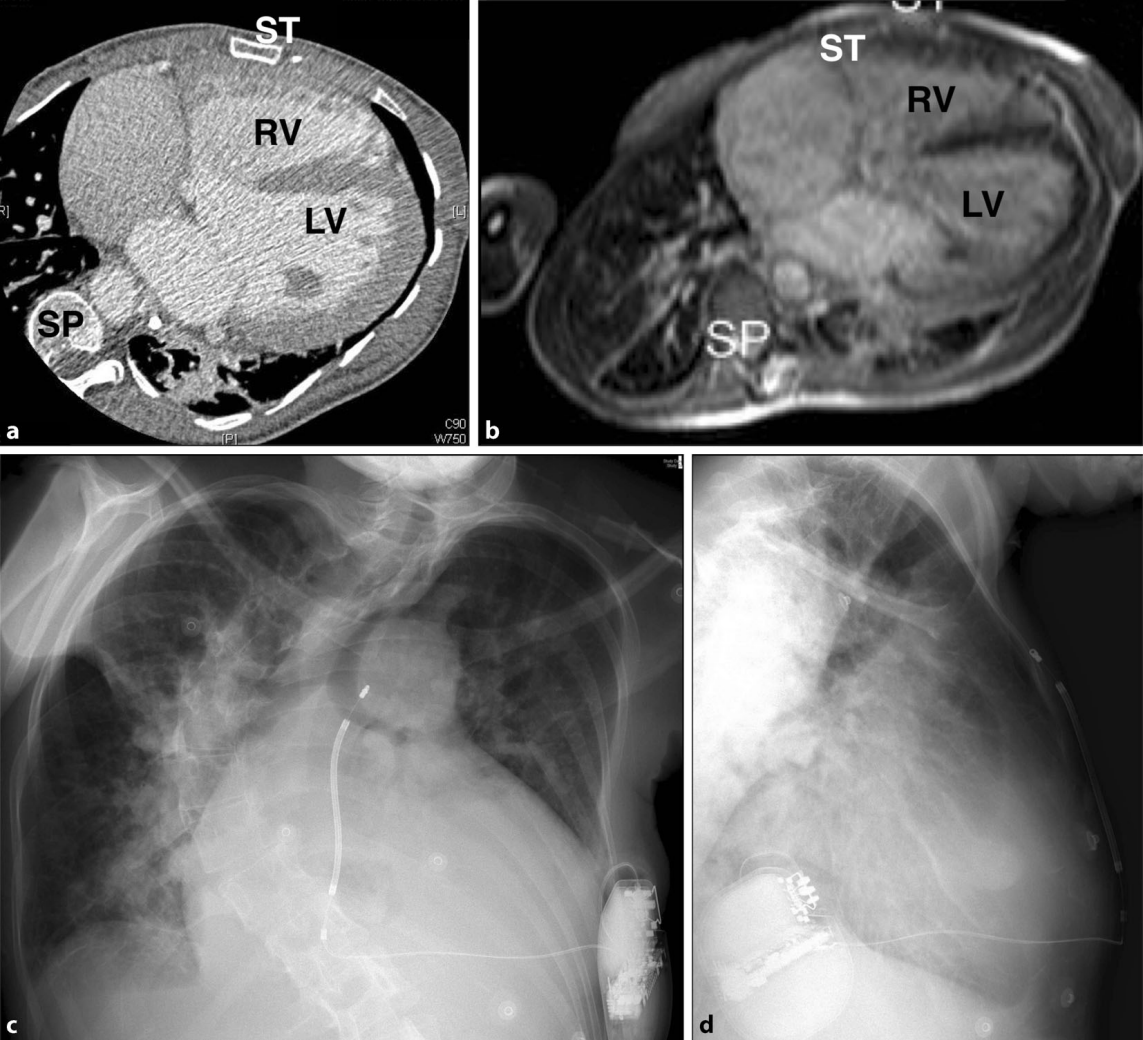
Patient with severe scoliosis, pulmonary atresia, ventricular septal defect and major aortopulmonary collateral arteries. **a** Computed tomography and **b** Balanced steady state free precession (b‑SSFP) magnetic resonance image. **c**  Anteroposterior and **d**  lateral radiographs demonstrating subcutaneous implantable cardioverter-defibrillator with excellent shock vector (subcutaneous coil has been placed to the right of the sternum, *RV* right ventricle, *LV* left ventricle, *SP* spine, *ST* sternum)

Early evidence of efficacy and complication rates is emerging, with relatively promising results [11, 12, 23, 33]. Initial concerns regarding sensing issues, inappropriate shocks and failure of conversion of malignant arrhythmias seem to have been broadly overcome with appropriate programming and placement but S‑ICD remains a technology in its early phases with limited use in the structural CHD population [23]. However, its implementation in patient groups with no venous access to the heart is attractive if pacing is not required (Fig. 4) and there is evidence of reduced lead failure rates compared to non-transvenous systems [32].

### Other solutions

**Fig. 5:**
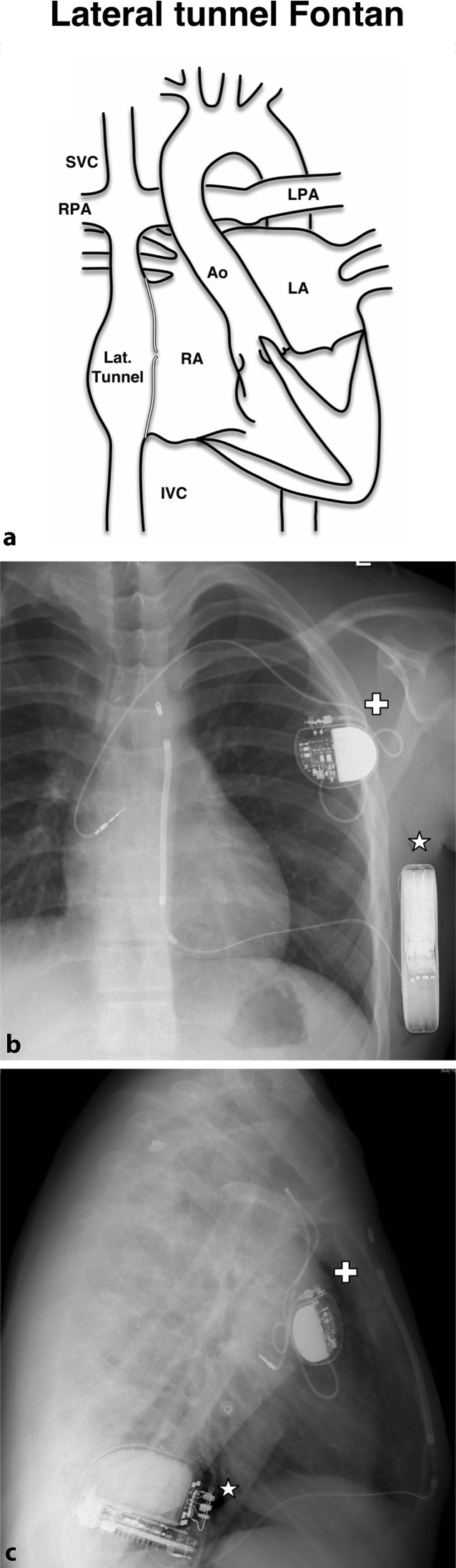
Diagram of subcutaneous implantable cardioverter-defibrillator and transvenous atrial pacemaker in patient with lateral tunnel Fontan (**a**). Posteroanterior (**b**) and lateral (**c**) radiographs demonstrating single chamber pacemaker (white cross) with lead to systemic venous portion of the right atrium and subcutaneous implantable cardioverter-defibrillator (white star). Figure adapted with permission from Chubb et al [7]

For some patients a hybrid approach may offer the best solution, combining transvenous with non-transvenous or S‑ICD systems, particularly where atrial pacing is required (Fig. 5). A transatrial approach (standard ICD lead placed directly into ventricle via atrial wall) has also been reported [5].

A further alternative is the wearable cardioverter-defibrillator (LifeVest®, ZOLL, Pittsburgh, PA) and the WEARIT-II registry provided a limited degree of evidence for efficacy in the CHD GROUP. Out of 2000 patients, 163 had CHD and a high therapy efficacy was reported in all groups [26]. Such a system may play a role in decision-making prior to more permanent system implantation or a final decision to withhold ICD therapy but successful clinical implementation would be highly dependent on compliance.

### Configuration

The increased frequency of non-transvenous system implantation, in the context of abnormal cardiac orientations, presents a unique challenge in selecting the optimal ICD configuration. Using MRI derived heart-torso models, attempts have been made to model electrophysiological responses to simulated defibrillation and these pipelines have yielded promising early results [35]. Clinical implementation remains several steps away but there is likely to be a role for such technologies in the future.

## Long-term management

### Follow-up

Remote monitoring and device automaticity have greatly facilitated follow-up, with many centres aiming for approximately 6‑monthly face-to-face follow-up for patients once systems are established. Guidelines for the level of dependency that can be placed upon remote monitoring systems remain to be determined but from a practical perspective the majority of patients with CHD will require on-going general cardiology review, in addition to device management.

#### Defibrillation threshold testing

There is a general consensus for defibrillation threshold (DFT) testing at new implantation and lead revision but the approach to testing at generator change and general surveillance is more varied. A study by Stephenson et al. in 2005 showed that routine DFT testing in asymptomatic patients is unlikely to lead to clinical changes and testing should instead be guided by clinical indications, such as change in lead position and sensing/pacing characteristics [37]. The latest ESC guidelines suggest that periodic DFT testing of non-transvenous ICD systems should be considered in young children (class IIa, level C recommendation) but the specifics are deliberately vague [42].

### Cardiac magnetic resonance imaging

The role of MRI for surveillance and investigation of CHD management continues to increase and non-cardiac MRI is frequently indicated in the maturing adult CHD population. There is therefore a role for MRI conditional systems and the majority of manufacturers now provide MRI conditional solutions for ICDs (Medtronic: SureScan®, St Jude: MRI Ready, Biotronik: ProMRI®, Boston Scientific: ImageReady®). Selected MRI conditional generators must be combined with MRI conditional leads to provide the necessary static magnetic, gradient magnetic and high frequency (RF) field safety. Manufacturers all provide individual guidelines but generally imaging should be delayed until a minimum of 6 weeks following implantation and height restrictions may apply if imaging is to be performed on-label. There are also differences in MR conditionality: full body scan conditionality enables cardiac MRI but some systems have exclusion zones precluding cardiac MRI particularly at 3 Tesla.

**Fig. 6:**
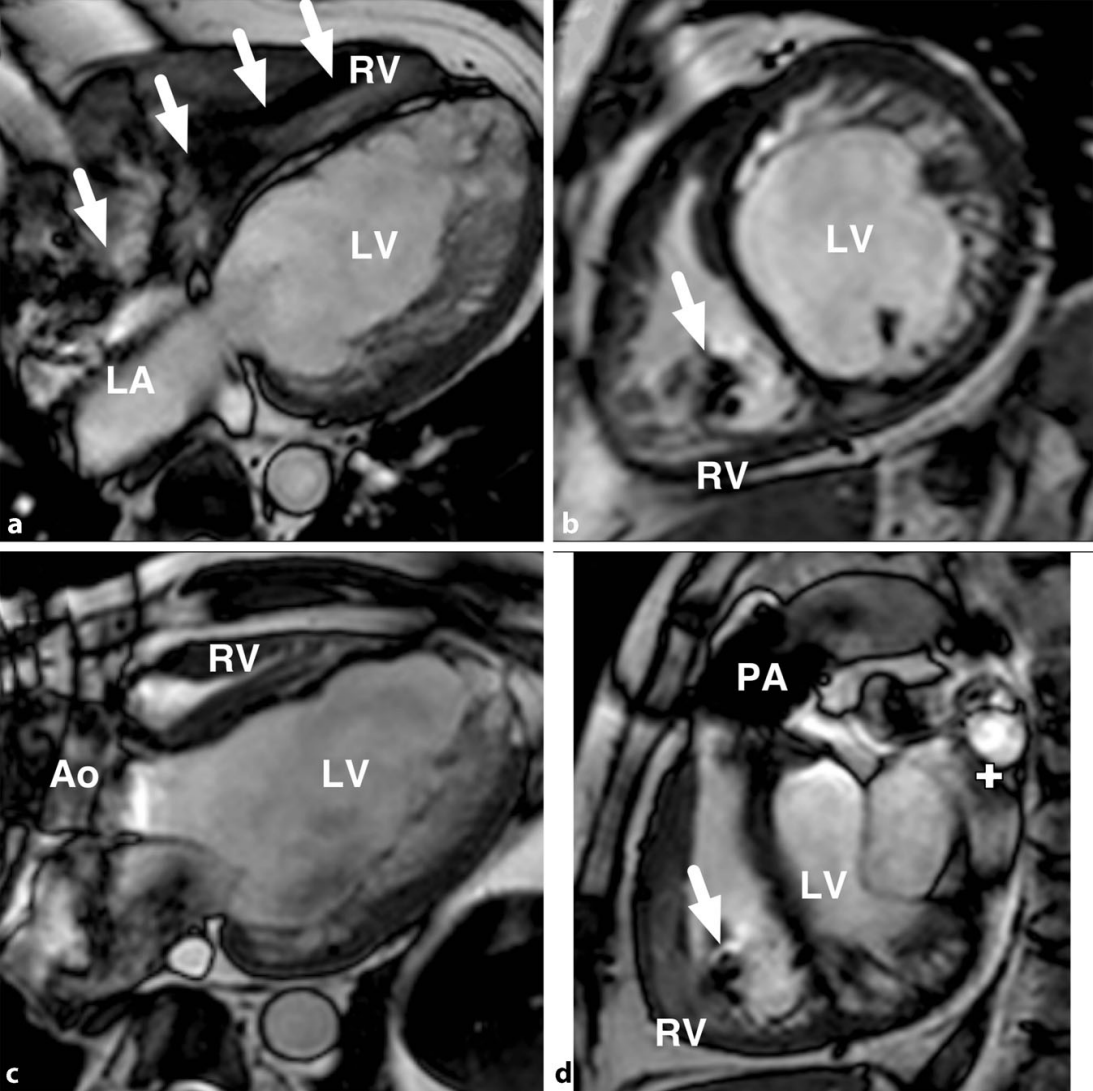
Cardiac magnetic resonance imaging in a patient with congenital aortic stenosis (status post-Ross procedure) and magnetic resonance-conditional implantable cardioverter-defibrillator, demonstrating typical results for balance steady state free precession (b‑SSFP) cine imaging. Lead position is indicated by *white arrows* and the ring artefact related to the generator is seen at the top left of panels (c) and (d). **a** four chamber view, **b** short axis, **c** three chamber view, **d** right ventricular outflow tract view. *RV* right ventricle, *LV* left ventricle, *Ao* aorta, *LA* left atrium, *PA* pulmonary artery

Despite the MRI-conditionality of the devices, substantial imaging artefacts related to the generator and leads should be anticipated (Fig. 6). The extent of these artefacts is unpredictable but generally relate at least in part to proximity of the generator to the heart and specific MR sequences may be more robust than others. Consideration to imaging prior to implantation should continue to be made, even if an MR-conditional system is to be implanted and continuous cardiac monitoring should be employed throughout scanning as ICD therapies are switched off in MRI scan modes.

### ICD-related complications

#### Inappropriate shocks and ICD programming

Vehmeijer et al. [39] looked at 518 patients with adult congenital heart disease (ACHD) across 16 studies and inappropriate shocks were reported in 25 % of patients over approximately 3.8 years of follow-up. In children the figures were similar with Berul et al. reporting inappropriate shocks in 70 out of 290 children (24 %), albeit in the context of a slightly lower adult inappropriate shock rate (14 %, *p* <0.05) [3].

A minority of these events can be traced back to lead failure or oversensing but up to 85 % are related to supraventricular tachycardia (SVT) (including sinus tachycardia) [39, 41]. The ICD programming therefore plays a key role in the minimization of shocks. A number of approaches can be adopted and the task of balancing over-treatment against delaying therapy is a delicate one [22]. Device technologies continue to evolve, but individual tailoring of devices remains crucial to reduce inappropriate therapies. This should be performed in the knowledge of pre-existing arrhythmia characteristics, but also noting that these very characteristics may vary widely from episode to episode.

Faster cut-off rates, especially in children, will reduce the chance of SVTs falling into the therapy zone. In some cases where there is significant overlap between appropriate sinus tachycardia and ventricular arrhythmia rates, there is a role for beta-blockade, which may also reduce the incidence of atrial arrhythmias [4]. Relatively slow intra-atrial re-entrant arrhythmias predispose to 1:1 conduction, particularly in children, and may necessitate the implementation of manufacturer-specific discriminator algorithms, such as morphology and onset analysis; however, the use of dual chamber, rather than single chamber, systems has not been shown to provide added protection from inappropriate shocks in this patient group [27]. Catheter ablation should be used when possible to reduce inappropriate shocks.

ATP employs burst pacing algorithms to interrupt re-entrant tachycardia in the atrium or ventricle, either of which may deteriorate to a rhythm precipitating loss of cardiac output. Non-CHD trials have demonstrated the safety, efficacy and patient acceptability of these programming options [30]. ATP should also be employed in the CHD population, where they have been shown to be successful in terminating a high proportion of VT episodes [14].

#### Other complications

The rate for other complications in the CHD population is higher than that seen in large non-CHD cohorts, with complication rates around 25 % over 3.5 years, compared to 14 % in the non-CHD population over a similar follow-up period (Sudden Cardiac Death in Heart Failure Trial, SCD-HeFT) [2, 39, 41].

The most common complication is lead failure or dislodgement, which is likely to represent a combination of anatomical complexity compromising lead positioning and the active lifestyle of the predominantly young population. In the large PLEASE study, Atallah et al. clearly demonstrated the association of lead failure with younger implantation age and Sprint Fidelis leads, with an overall actual yearly failure rate of 2.3 % for non-Fidelis leads [1]. Lead and generator infections are much more rare but present significant management problems and morbidity.

### Lead extraction

In general, ICD lead extraction appears to be feasible, reasonably safe but technically difficult [1, 6, 29]. In the PLEASE study, lead extraction was achieved for 143 leads, without mortality but with a 4.3 % rate of major complications [1]. Half of all lead extractions required advanced tools, including locking stylets and powered sheaths and technical skill and operator experience are an important codeterminant of procedural success.

### Quality of life

ICD therapy undoubtedly has life-saving potential when deployed in the correct patient group; however, a significant number of patients have psychological consequences, particularly amongst those that have experienced shocks. In large cohort studies of non-CHD patients, there was no overall difference in quality of life for those randomized to ICD therapy. However, in a prospective multicentre study from Alliance for Adult Research in Congenital Cardiology it was noted that CHD ICD recipients experienced a high level of shock-related anxiety, with associated impact on sexual function in both sexes [8]. In parallel, studies of children with ICD have also demonstrated a significant impact on quality of life [9]. The mitigation of these adverse effects on quality of life, through measures such as psychotherapy, remains to be established.

## Conclusion

The use of ICD therapy in CHD is a life-saving treatment in appropriately selected patients. The majority of systems are implanted transvenously but novel and innovative techniques may be required and in some cases a non-transvenous solution may be most appropriate. Complication rates, including inappropriate shocks and lead failures are higher than those seen in the non-CHD population and life-long exposure to these risks should be carefully weighed up against the benefit the patient is anticipated to derive from the system. Large and inclusive registries, with good follow-up are needed to provide robust data on patient management, including indications for primary prevention ICD implantation. This, combined with evolving generator, lead and programming technologies, should continue to improve ICD management for these complex patients.
